# A case of hydatid cyst in biceps femoris

**DOI:** 10.1016/j.ijscr.2019.11.056

**Published:** 2019-12-03

**Authors:** Mirsalim Seyedsadeghi, AmirAhmad Arabzadeh, Afshin Habibzadeh

**Affiliations:** a Department of Surgery, School of Medicine, Ardabil University of Medical Sciences, Ardabil, Iran; b Department of Internal Medicine, School of Medicine, Ardabil University of Medical Sciences, Ardabil, Iran

**Keywords:** Biceps femoris, Hydatid cyst, Iran

## Abstract

**Introduction:**

Hydatid cysts primarily involve the liver and lung, but could be presented in any site of the body including the muscles which is very rare.

**Presentation of case:**

We report a 50-year-old woman with hydatid cyst in biceps femoris presenting as growing painful mass. There was also a small cyst in the liver. The cyst was completely excised and patient was treated with albendazole 15 mg/kg daily before and after surgery. The patient was symptom free during the three months follow-up.

**Discussion:**

Hydatid cyst should be considered as differential diagnosis of any growing mass or cyst in the body in the endemic areas.

## Highlights

• Hydatid cysts primarily involve the liver and lung.
• Hydatid cysts could be presented in any site of the body including the muscles which is very rare.
• Hydatid cyst was observed as growing mass in the biceps femoris.
• Hydatid cyst should be considered as differential diagnosis of any growing mass or cyst in the body in the endemic areas.

## 1 Introduction

Hydatid disease is a parasitic infection mostly caused by larvae of the Echinococcus granulosus [1,2]. It is common in sheep-raising countries [3] including the Middle East. The disease is endemic in Iran, with most cases reported from north western region [1,2].

Due to the benign and asymptomatic progression of the hydatid cyst, it usually presents in its advanced form [4]. Although the liver and lungs are the most involved organs, but different organs could be involved by the hydatid cyst [1,2]. Musculoskeletal involvement is very rare and involvement of the biceps femoris is much rarer [[3], [4], [5], [6], [7], [8]]. Due to the high lactic acid levels, the muscles usually provide poor environment for the parasite and makes it an unusual place for presentation [5].

Here we report a case of hydatid cyst in biceps femoris presented as a painful mass in the back of the right thigh. The work has been reported in line with the SCARE criteria [9].

## 2 Case presentation

A 50-year-old woman presented with the complaint of pain in the back of right thigh for 2 month to our institute in 2019. The pain was worsened with daily activity and get better with rest. The patient had no history of medical disease. In physical examination, there was a swelling 7*7 cm in the middle of the back of the right thigh with no erythema, tenderness or warmness. Neurologic and other examinations were normal.

**Fig. 1:**
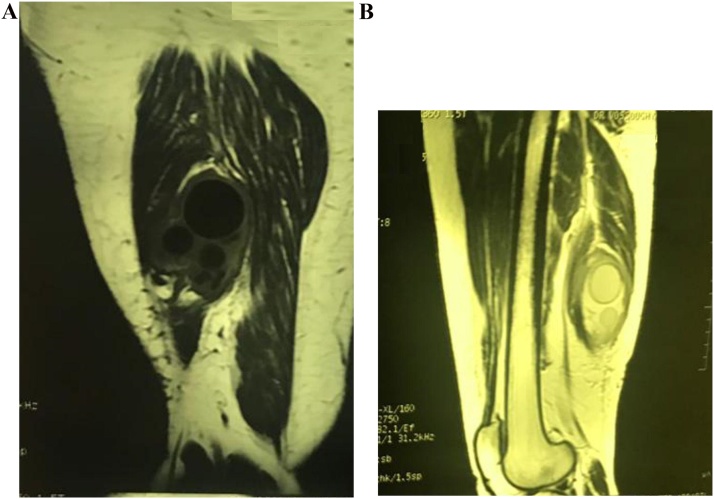
MRI showing cystic swelling in biceps femoris in T1 (a) and T2 (b) view.

Chest x-ray was normal. Ultrasonography of the abdomen showed 12*19 mm cystic lesion with multiple fine septations in the right lobe of the liver. US of the right thigh showed 8 cm mm cystic lesion in the back of the right thigh with multiple septations indicative of daughter cysts. Magnetic resonance imaging of the right thigh also showed multi locular muscle mass inside the semimebranous muscle with mass effect on semitendinous and long head of biceps femoralis. There was hyposignal intensity in T1 and hypersignal intensity in T2 with small round shape cyst with double layer wall inside of it suggestive for hydatid cyst (Fig. 1a,b). Other organs were otherwise normal.

**Fig. 2:**
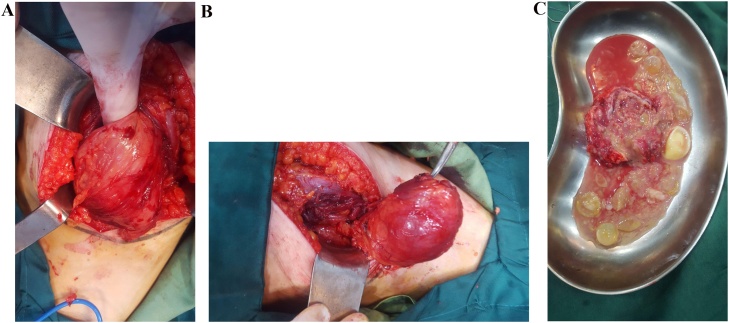
Preoperative gluteal hydatid cyst (a & b) and postoperative specimen including daughter cysts after cyst was opened (c).

Due to the intense and persistent pain, the patients was subjected to surgical cystectomy. Albendazole 15 mg/kg daily were administered for two weeks prior to surgery. Under spinal anesthesia, en block surgical excision of the mass was performed with care without perforating the cyst wall (Fig. 2a,b,c). The liver cyst was not surgically treated due to its small size. Post-operative period was uneventful. The patient was discharged on albendazole 15 mg/kg daily for two months course. The patient was free of symptoms with no recurrence during the regular visits in the next three months after surgery follow-up and in the final visit at third months.

## 3 Discussion

Hydatid cyst is endemic in most parts of the world, especially sheep- or cattle-raising regions and considered as a health problem in developing countries due to the lack of the appropriate programs to prevent its transmission [10]. Liver and lungs are the common site of invasion for the cyst; however, the cyst can appear in different sites of the body. Muscle involvement by the hydatid cyst is very rare because of the high lactic acid levels that makes them unsuitable environment for the parasite [5,10]. Muscle involvement could be primary or concomitant with liver or lung involvement. Among muscles involved, there are some reports of gluteal [2], thigh [4], biceps femoris [3,[5], [6], [7], [8]] and biceps brachii [11]. Besides our case, there are only five previous case report reporting the hydatid cyst in the biceps femoris [3,[5], [6], [7], [8]].

The unusual sites of presentation may lead to nonspecific symptoms and the disease is usually diagnosed in its advanced stages. The common presentation in muscle involvement is painful muscle mass and thus is usually mistaken with soft tissue tumors. Although difficult to diagnose preoperatively, it is important to rule out hydatid cyst before biopsy to prevent cyst leakage and consequent anaphylaxis [10].

The common imaging modalities are ultrasonography, computed tomography to identify the characteristic findings of the cyst, but for muscle, MRI is more sensitive [2]. Which in our case, it could define the exact location and involvement of different muscles.

Total surgical excision is recommended for hydatid cysts more than 5 cm, especially when they are in the unusual sites [2]. Treatment with antihelminthic drugs is recommended for small size cysts and for all cases undergoing surgery, prior and after operation.

## 4 Conclusion

Hydatid cyst should be considered as differential diagnosis of any growing mass or cyst in the body in the endemic areas.

## Sources of funding

No funding source to report.

## Ethical approval

This case report was exempt from ethical approval in our institution.

## Consent

We have the patient’s consent for publication of the submitted article and images.

## Author contribution

MirSalim SeyyedSadeghi and AmirAhmad Arabzadeh conceived the idea for the study. All authors were involved in data collection. Afshin Habibzadeh wrote the first draft of the manuscript. All authors edited and approved the final version of the manuscript.

## Registration of research studies

NA.

## Guarantor

MirSalim SeyyedSadeghi and AmirAhmad Arabzadeh.

## Provenance and peer review

Not commissioned, externally peer-reviewed.

## Declaration of Competing Interest

We have no conflicts of interest.
